# Responsibility-Sharing in the Giving and Receiving of Assessment Feedback

**DOI:** 10.3389/fpsyg.2017.01519

**Published:** 2017-09-06

**Authors:** Robert A. Nash, Naomi E. Winstone

**Affiliations:** ^1^Department of Psychology, Aston University Birmingham, United Kingdom; ^2^Department of Higher Education, University of Surrey Guildford, United Kingdom

**Keywords:** feedback, assessment, student engagement, teaching excellence, culture, sustainability

## Abstract

Many argue that effective learning requires students to take a substantial share of responsibility for their academic development, complementing the responsibilities taken by their educators. Yet this notion of responsibility-sharing receives minimal discussion in the context of assessment feedback, where responsibility for enhancing learning is often framed as lying principally with educators. Developing discussion on this issue is critical: many barriers can prevent students from engaging meaningfully with feedback, but neither educators nor students are fully empowered to remove these barriers without collaboration. In this discussion paper we argue that a culture of responsibility-sharing in the giving and receiving of feedback is essential, both for ensuring that feedback genuinely benefits students by virtue of their skilled and proactive engagement, and also for ensuring the sustainability of educators' effective feedback practices. We propose some assumptions that should underpin such a culture, and we consider the practicalities of engendering this cultural shift within modern higher education.

In higher education, as in many other walks of life, the delicate processes of giving and receiving feedback are challenging to negotiate. The essence of this challenge has been captured perfectly by Stone and Heen (2014, p. 3), who observed:

“Interesting. When we give feedback, we notice that the receiver isn't good at receiving it. When we receive feedback, we notice that the giver isn't good at giving it.”

Who is to blame when feedback does not improve learning, does not enhance student satisfaction, or indeed does not get used at all? As Stone and Heen's observation implies, many students can seem quick to blame educators for giving poor feedback, whereas many educators can seem equally quick to blame students for engaging poorly with the feedback. These conflicting perspectives can lead to a sense from both parties that the feedback process is futile. In this discussion paper we argue that if our aim is to ensure feedback has a strong impact, then we must find ways to foster a culture of shared responsibility between educators and students.

## Responsibility in higher education

To begin considering the importance of shared responsibility in the context of giving and receiving feedback, it is valuable first to consider the climate of responsibility-sharing within higher education more broadly. In today's higher education systems around the world, there are growing concerns over the perceived movement toward “consumerist” approaches to learning and teaching (Bunce et al., in press). These concerns center on the notion that students in higher education are increasingly being positioned (and often are positioning themselves) as the passive recipients or customers of a service that, in more and more cases, they have paid considerable sums to receive. Many fear that this consumer model of education leads students to become detached from their personal responsibilities in the learning process, and to an unrealistic accountability on educators to deliver results and to resolve all challenges (McCulloch, 2009). Indeed, these fears were somewhat validated by a recent survey of the attitudes and academic performance of 608 UK undergraduate students (Bunce et al., in press). In that study, the students' learner identities—including their attitudes such as enjoying and valuing learning, and behaviors such as attending classes and engaging with reading—strongly predicted their academic performance. But more importantly, this relationship was statistically mediated by the students' consumer identities: students with weak learner identities tended to score highly on measures of consumer identity, and in turn, performed less well academically. The negative association between consumer identity and academic achievement serves as a strong cautionary note, underscoring wider concerns about the fundamental importance of responsibility taking in education.

Against the backdrop of a movement toward consumerism fueled by wider socio-political and economic changes, a contrasting movement has been underway in educational theory and best practice—one that seeks to place greater value on student-centered approaches. For example, Cannon and Newble (2000, p. 16) write that a valuable approach should “emphasize student responsibility and activity in learning rather than what the teachers are doing.” Others argue that although the student needs to take on responsibility and autonomy within the learning process, the key factor is interdependence, rather than the student being completely independent or dependent (e.g., Lea et al., 2003). Similarly, McCulloch (2009, p. 178) proposes a “co-production” alternative to the consumerist approach, which reduces the emphasis on the role of the educator, and apportions greater responsibility to the student by recognizing that “both student and university bring resources to the educational process, and that both make demands and levy expectations on each other during that process.”

These approaches all share a commonality in agreeing that high-quality teaching alone is insufficient for delivering high-quality learning. The notion of needing a shared responsibility between educators and students has long-standing support in the educational literature. Biggs (1999), for example, argues that having a complete model of teaching competence requires us to focus not only on the behavior and responsibilities of the educator, but also on those of the student. He encapsulates this argument with an excellent quote from Thomas Shuell, who wrote:

“It is helpful to remember that what the student does is actually more important in determining what is learned than what the teacher does”. (Shuell, 1986, p. 429, as quoted in Biggs, 1999).

Considered together, these diverse perspectives show substantial consensus that students' progress in higher education can be facilitated by, or indeed is wholly contingent on, their ability and willingness to share responsibility for their learning. With this point in mind, it stands to reason that similar kinds of responsibility-sharing should be beneficial within the specific context of receiving assessment feedback. This, as we will argue shortly, is undoubtedly the case. But to what extent do current learning cultures within higher education encourage or require students to take responsibility for how they seek and implement feedback?

## Responsibility in the context of feedback

Feedback is essential to learning: we cannot reasonably expect students to develop academic skills and understanding without them receiving such crucial information and direction (Black and Wiliam, 1998; Hattie and Timperley, 2007). Yet within higher education, feedback is the most prominent source of students' dissatisfaction with their programmes of study. In the UK for example, data from the annual National Student Survey (NSS) have consistently shown that even though almost all university students are satisfied overall with their course, only around three-quarters are satisfied with their experiences in the domain of assessment and feedback (Higher Education Funding Council for England, 2016).

In response to this perennial problem, many institutions have placed responsibility squarely with educators for improving the quality of the feedback they give to students. In many cases, these efforts have involved urging educators to provide more and more detailed feedback to students, often doing so by completing ever more structured and intricate pro formas (e.g., Case, 2007). Yet the NSS data show that despite these efforts, only relatively modest improvements in satisfaction with feedback have transpired over the space of many years (Higher Education Funding Council for England, 2016). These weak effects probably come as little surprise to experts on assessment and feedback, who identify these kinds of solution as symptomatic of what is often termed the “transmission view” of feedback (e.g., Nicol, 2010).

The transmission view conceives of assessment feedback as a process whereby information and advice are delivered in a linear manner from expert to novice. The linear structure of this process, critically, implies relatively little responsibility on students' behalves for making feedback effective. Rather, whenever feedback processes are judged to have been unsatisfactory or ineffective, the cause is typically attributed to some shortcoming in the quality or timeliness of the information that was transmitted. Evidence for the dominance of the transmission perspective can be gained from even a cursory glance through many higher education institutions' policies and guidelines on feedback, wherein recommendations can focus entirely on what academics should do, and how their feedback comments should be phrased. Indeed, one might argue that the survey items in the NSS also reinforce this transmission perspective, by placing sole emphasis on the active delivery of feedback information to students, and the passive receiving of this information by students (see Table 1, and Nicol, 2010). The items were amended in 2017, although these amendments appear to have fallen short of establishing a move away from a transmission-centered discourse.

**Table 1:** Assessment and Feedback items in the 2017 UK National Student Survey (NSS).

1	The criteria used in marking have been clear in advance.
2	Marking and assessment has been fair.
3	Feedback on my work has been timely.
4	I have received helpful comments on my work.

Despite its ubiquity, scholars in the area of assessment and feedback have called for the “old paradigm” transmission view to be replaced by a “new paradigm” in which the feedback process is instead seen as two-way dialogue (Nicol, 2010; Carless, 2015). For example, Carless (2006, p. 192) conceptualizes feedback as “a dialogic process in which learners make sense of information from varied sources and use it to enhance the quality of their work or learning strategies”. This conceptualization is valuable because it emphasizes the active role necessarily played by the student in the feedback process, invoking a partnership of responsibility between educator and student rather than the responsibility resting solely with the educator. In a similar vein, Nicol (2010) speaks of the importance of educators and students “sharing the burden” in this process, and the UK's Higher Education Academy (2012) advocates placing greater emphasis on students' engagement with feedback. Giving ever more detailed feedback, they suggest, can lead to unsustainable workload pressure on educators, whilst often having minimal impact on students' learning (for similar arguments from the schools sector, see Independent Teacher Workload Review Group, 2016). Deeley and Bovill (2017) argue that a staff—student partnership approach in general can raise students' intrinsic motivation. However, they caution that this approach is often perceived as more difficult to achieve in the area of assessment and feedback relative to other areas of learning and teaching, because historically the responsibility for assessment and feedback has been seen as resting solely with educators. One of the key implications of a new paradigm perspective, then, is that although the effectiveness of feedback still rests partly on the quality and timeliness of the information that is communicated, it also, critically, rests on how well and how proactively the student engages with this information.

## Students' engagement with feedback

There is an implicit perception held by many students and educators, that improvements in students' skills and performance will occur simply by virtue of feedback being provided (Crisp, 2007). But in reality, simply receiving feedback—no matter how high in quality—can never lead students to improve unless they actively receive, digest, and act upon it—what we have previously termed *proactive recipience* of feedback (Winstone et al., 2017, in press). Perhaps because the transmission view of feedback has been so ubiquitous in higher education, the student's role in engaging with feedback has often been ignored or under-represented in the research literature, leading to what Burke (2009) called a “blind spot” in our understanding of the issue.

Fortunately, the tide is beginning to turn on this matter, and in particular the work by Margaret Price and colleagues has been influential in shifting the spotlight of attention toward engagement (e.g., Handley et al., 2011; Price et al., 2011). In fact, their body of work challenges our very understanding of engagement with feedback. Handley et al. (2011), for instance, caution against misinterpreting students “doing time” with feedback as evidence of their strong engagement. They argue that a student who merely skim-reads their feedback, without taking further action, is doing little more than paying lip service. More important, they argue, is what they term students' “readiness to engage” with feedback: their attitude of commitment and willingness to expend effort on implementing advice, rather than just being willing to receive it. Indeed, whereas readiness to engage may be an important precursor to proactive recipience, it is not necessarily the only one. In a systematic literature review, we identified four broad types of skills that have been assumed to play roles in supporting students' proactive recipience: self-appraisal, assessment literacy, goal-setting and self-regulation, and motivation (Winstone et al., 2017). Supporting students to develop these skills should in principle help them to develop as proactive recipients of feedback.

How convincingly do students demonstrate proactive recipience? At first glance, the higher education literature paints a bleak picture. There we find numerous accounts of poor—and in some cases entirely absent—engagement with feedback. At a basic level, we find reports of students failing to even collect their written feedback (e.g., Sendziuk, 2010; Scott, 2014), and evidence that they are wholly aware of their shortcomings in this regard, as illustrated by a student in one study who commented “*I don't really take much notice of [feedback] to be honest*” (Rae and Cochrane, 2008, p. 222). Other reports suggest that students merely skim-read the written comments that their educators provide (Gibbs and Simpson, 2004), and that for many students, even those who read beyond this cursory level, their initial reading of the written feedback represents the end of their engagement with it (Robinson et al., 2013). Additionally, studies report finding little evidence that feedback is actually put into practice in students' future work (e.g., Crisp, 2007).

But findings such as these are firmly at odds with Higgins et al.'s (2002, p. 59) characterisation of students as “conscientious consumers” who are eager to receive feedback, and show strong engagement with it. In their survey, 82% of first-year undergraduate students in business and humanities disciplines agreed with the statement “I pay close attention to the comments I get” (Higgins et al., 2002, p. 57). And like Higgins et al., many other groups of researchers find cause for optimism. For instance, Zimbardi et al. (2017) used learning analytics to track first- and second-year undergraduates' engagement with feedback. The authors found not only high levels of engagement among their students, but also evidence that those students who engaged for longer durations typically achieved larger grade increases on their subsequent assignments. In qualitative studies, many student participants reveal considerable insight into the benefits of engaging with feedback. For example, one student in Orsmond et al.'s (2005, p. 375) interviews stated “*When reading feedback it makes you realize what you could have done, rereading an essay with the feedback in mind helps you to see work in a different light”*. Likewise, in Wingate's (2010, p. 529) interviews, one student stated “*I looked at all the mistakes like clumsy expressions, and I thought this time I really need to think what I am going to say. So I got a book from the library, called “Writing at University”, or so, and started reading that on the train and everywhere where I could read.”*

To gain our own sense of this issue, we asked 96 of our psychology undergraduates to complete a short online survey about the summative feedback they receive from their lecturers (see Winstone and Nash, in press, for further details). One of the questions we asked was “When you receive feedback on a piece of coursework, what do you do with the feedback?”. Students were invited to respond in open text format. Their responses revealed considerable variation in the depth of their engagement. There was some reason for optimism, with some students demonstrating deep engagement with their feedback. One, for example, wrote:

“I highlight the bits I think will be most helpful, and write them on post-it notes ready for further work. I focus on improvements which I can make, and try to see my downfalls and strengths”.

Unfortunately though, relatively few of the students' responses gave indications of going beyond shallow and cursory reading of feedback information. The following responses illustrate this problem:

“I tend to skim read the feedback sheet, mainly look at the comments written on the actual piece of coursework.”

“I keep all feedback but rarely look at it after the day I receive it despite good intentions.”

“I often give it a glance over when I first receive it, but hardly ever go back to it when doing another assignment of a similar nature even though I know it may be helpful!”

Piecing these varied research findings together, it is clear that not all students recognize the necessity of engaging proactively with feedback, and that even the efforts of those who do are not always adequate or effective. One might, at this point, conclude that the case is therefore closed: clearly, students themselves are to blame for why feedback so often fails to make a difference. This conclusion, we would argue, is neither helpful in correcting the problem, nor is it correct. To see why, we must consider what barriers exist that limit or prevent students' effective engagement with feedback.

## Barriers to engaging with feedback

Based on a small-scale literature review, Jonsson (2013) proposed five key issues that limit students' usage of the feedback information they receive: (1) the advice may be insufficiently useful or useable; (2) feedback may be too generic, non-specific, or lacking in individualisation; (3) the tone of feedback may be too authoritative; (4) students may be unaware of the strategies they could use to implement feedback; and (5) the language used in feedback may be difficult to understand. These proposed barriers give us some considerations that educators might consider with regard to the format and content of their feedback. Yet it is noteable that with the exception of (4), all of these explanations attribute failures in proactive recipience to shortcomings of the feedback information itself—something that, as we have argued above, resonates with a transmission rather than dialogic view of feedback, and appears to place responsibility squarely with educators.

Is it the case, then, that educators alone could in fact solve most of the issues with students' engagement with feedback, simply by paying greater attention to the tone and content of their feedback messages? We strongly doubt it. Rather, we believe that the five barriers identified in Jonsson's (2013) review underestimate the true breadth of barriers that can exist in this context. In a recent study of this issue, we conducted activity-oriented focus groups with undergraduate psychology students, in which we elicited participants' reflections on how they use feedback, but paid principal attention to their spontaneous discussions of what *prevents* them from using feedback (Winstone et al., in press). By scrutinizing the dialogue from these focus groups, we conducted a thematic analysis that revealed four broad kinds of psychological barrier, as follows:

### Awareness

One reason why students apparently fail to engage with feedback is that they simply cannot understand it, do not know what it is for, or perhaps do not even realize that they have received feedback. Many researchers have observed that educators and students are often severely misaligned in their understandings of the definition and purpose of feedback. In work by Adcroft (2010), for example, educators and students disagreed even on how frequently feedback was being given—88% of educators believed they were giving frequent feedback, but only 12% of students agreed. Moreover, Jonsson's (2013) review highlighted that students do not always understand the terminology and academic jargon used within feedback. One student in our focus groups exemplified this point, stating “*sometimes on the feedback, it's just a lack of understanding of what it means…that holds you back from using it*”.

### Cognisance

A second reason is that students can lack knowledge of the opportunities available for them to implement their feedback effectively, or—as identified in Jonsson's (2013) review—can lack knowledge of strategies they could possibly take as a means to help them act upon the feedback. Whereas it is easy to take for granted that students know what to do with feedback, evidence suggests that this is not routinely the case. For example, Weaver (2006) showed that only 50% of students surveyed had ever received guidance on how to use feedback; similarly, Burke (2009) reported that only 39% of student respondents to her survey had received guidance prior to starting university on how to use feedback. In short, students might know that there is a particular skill they need to improve, but they must also know how to enact that change, what steps to take, and how to affirm that those efforts have been successful.

### Agency

A third reason is that students can feel insufficiently equipped to deal with feedback, or feel that doing so would be futile. In some cases, this lack of agency can arise because students believe that the skills or qualities they are being advised to develop are fixed, rather than modifiable through effort. For example, despite repeatedly receiving criticism about their writing style, they may believe that this style is something intrinsic to themselves and therefore impossible to address. Students may also perceive that their prior attempts to respond to feedback have failed to “pay off” in terms of leading them to see enhancements in their performance and/or grades over time (Winstone et al., in press). Yet another common cause of limited agency to implement feedback can arise as a byproduct of the common modular structure of many degree courses, wherein students can find it incredibly difficult to see a transferability of advice from one assessment or module to the next (Orsmond et al., 2005; Jonsson, 2013).

### Volition

Finally, students can simply lack the motivation and enthusiasm to engage with feedback, being unprepared to invest the time or effort. Doing so requires the “readiness to engage” that Handley et al. (2011) have described, and a further “commitment to change” (Bing-You et al., 1997, p. 43), yet we found many of the student participants in our focus groups quite ready to acknowledge that these are not typically their priorities. Likewise, many academics perceive students' priorities similarly, reporting that students lack intrinsic motivation, and seek to do the minimum needed to attain a particular grade (King and Bunce, under review). Students' apparent apathy toward feedback information can in some cases be attributed to their primary interest in grades rather than in understanding their performance (Hounsell, 2007), and in other cases attributed to avoidance of the strong emotions that anticipating and receiving feedback can evoke (Higgins et al., 2002).

## Removing barriers to proactive recipience

Having identified a number of conceptual and specific barriers to engaging with and implementing feedback, one might ask: Whose responsibility is it to remove or mitigate these barriers? Based on Stone and Heen's (2014) quote at the start of this paper, we might predict that students would typically believe it is their educators' responsibility, whereas educators would typically believe it is their students' responsibility. Is this the case? In our survey of psychology undergraduates, we asked them “What might be done, or what might you do, to encourage you to make better use of the feedback you receive?” (Winstone and Nash, in press). Of the 89 responses we received to this particular question, 66% indeed focused solely on things their educators could do (e.g., “*Feedback should be more specific and detailed so I know exactly what to do when the next assignment falls*”). In other words, only 34% mentioned anything they, the students themselves, could do (e.g., “*Take better notes of the feedback, write it down, keep a list/tally of all the feedback I receive. This way I can go back to it when I feel I am falling back into old habits*”). This finding resonates with those from many other studies. For example, one student in research by Hounsell et al. (2005, p. 14) argued “*Maybe if they could give you more help with the assignments, and maybe a bit more feedback. You could have a monthly meeting with someone…to say to you…‘This is what was wrong with this assignment, this is what wasn't’”*.

To the contrary, when we asked 68 university lecturers and college teachers what they believed was the single biggest factor preventing their students from using feedback better, almost half foremostly blamed the students' weak motivation or volition (Winstone and Nash, in press). Indeed, when lecturers in Carless's (2006, p. 224) focus groups described factors that impede students' strong engagement with feedback, they emphasized students' strong focus purely on grades (“*Students don't use feedback for learning purposes; they only use it to see how well they've done, especially compared to others”*) and a lack of motivation to proactively seek feedback (“*[students] are not interested to meet their tutors to get feedback on how to improve their learning”*).

Together, these findings and the literature as a whole give the distinct impression of having reached an impasse. Many diverse barriers, we can see, stand in the way of students engaging proactively with the feedback they receive, and by extension, stand in the way of optimizing their skill development. But a culture of mutual blame between students and educators seems to prevent reasonable headway being made toward breaking down these barriers. As we argued at the start of this paper, when both students and educators mutually blame the other for the failings of feedback to make a difference, it is easy for both parties to conclude that the feedback process is futile.

**Figure 1:**
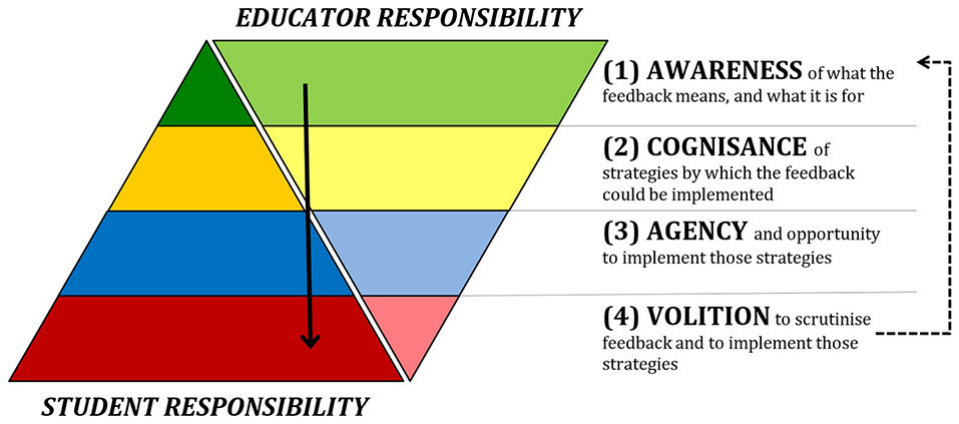
Distributions of responsibility for tackling barriers to proactive recipience.

Finding a resolution to this impasse, we believe, requires us to think more concretely about where different responsibilities could lie. We make several assumptions about the answer to this question, and illustrate these assumptions in Figure 1. The first assumption is that—mutual blame aside—both educators and students have essential roles to play; indeed, that overall these respective roles are approximately equal in significance, as represented by the respective sizes of the “educator” and “student” portions in Figure 1. Second, despite this equivalence of responsibility at the overall level, we assume the respective responsibilities of educators and students for resolving each individual kind of barrier are *not* equal. Rather, it is quite apparent from the discussion above that resolving certain barriers demands greater responsibility from students, whereas resolving others demands greater responsibility from educators. The relative sizes of the different levels within each portion of Figure 1 signify this second assumption.

Consider again the four kinds of barrier identified in our focus group research: awareness, cognisance, agency, and volition (Winstone et al., in press). Our third assumption is that when the barriers are sequenced in this particular order, they signify decreasing levels of responsibility on educators to resolve the issues, and increasing levels of responsibility on students. Considered in this way, it is perhaps unsurprising that both students and educators seem most readily to spot those barriers over which they themselves have the least control and responsibility. For instance, we have already noted that educators frequently identify students' volition to engage as being particularly critical. It seems reasonable to argue that students themselves must be primarily responsible for resolving this particular barrier. Certainly there are steps an educator might constructively take to encourage students to be motivated to engage, and to model the benefits of doing so. But it is the student who ultimately has greater power in this regard, and must be willing to co-operate and put in the “hard graft” required to implement feedback (Carless, 2015). Conversely, we have noted that students frequently identify as being particularly critical of various issues aligned with the barrier of awareness. They point out for example that the feedback they receive is insufficiently detailed, or that they do not understand it. In this case it seems reasonable to argue that educators must have primary responsibility for ensuring that the advice they give is clear and actionable. There are steps a student can take to enhance their understanding of feedback information and its intended purpose, but it is ultimately the educator who has greater power to ensure clear and effective conveying of meaning and purpose.

The fourth of our assumptions represented in Figure 1 is that the barriers follow a hierarchical, directional structure; that is to say, we must at least partly resolve those barriers at upper levels of the graphic before we can reasonably expect to resolve those at lower levels. For instance, it might be impossible to tackle a student's poor motivation to reflect on assignment feedback (i.e., a problem of volition), if this problem is largely underpinned by their belief that they will never again complete similar assignments, and hence that using the feedback would be pointless (i.e., a problem of agency). One might dispute this assumption by proposing that a student's volition, above all else, is the most fundamental ingredient of proactive recipience. We agree that volition is crucial, but would question the extent to which fostering volition is possible for a student who neither understands their feedback, knows anything they could do with it, nor believes they have the capability to improve. One implication of this fourth assumption, then, is that despite the approximately equal overall balance of responsibilities, educators are those with the greatest power to instigate changes in students' proactive recipience.

A fifth and final assumption illustrated in Figure 1 is that by increasing students' volition to engage with feedback, we can in turn create a virtuous cycle, making it easier both for students and educators to further break down the residual barriers at each level. Increased volition, for example, might lead students to invest greater time in reading and absorbing the written feedback they receive. It might also make them more likely to accept offers of dialogue: one study reported that only 31% of undergraduates who were offered discussions around their feedback actually took advantage of this offer, and few of these students showed evidence that they were highly familiar with the feedback they had received (Duncan, 2007). Increasing students' willingness to avail themselves of dialogue opportunities should, in turn, offer educators better opportunities to fulfill their own responsibilities.

How responsibility-sharing can be implemented in practice will undoubtedly vary across different disciplines and levels of education. Nevertheless, guided by the assumptions we have drawn, there should in all contexts be ways in which students and educators can work to remove the barriers to engaging with assessment feedback. For example, consider a common situation in which students receive written feedback after completing an essay. To overcome a lack of awareness of what feedback means and what it is for, the educator's responsibilities should include ensuring that the feedback they provide is clear, transparent, and linked to grading criteria. Students, on the other hand, have responsibilities including seeking clarification over the meaning of the feedback they receive. In overcoming barriers to cognisance, educators might build time in the curriculum for training students in the skills underlying the implementation of feedback, and avoid making assumptions about students' knowledge of strategies for acting on feedback. Students, for their part, might take responsibility for selecting which strategies to use in which situations, testing out new strategies, and deciding when to seek support beyond their usual “toolkit” of strategies.

In overcoming issues of agency, educators in this context might ensure that their comments are not too specific to one assignment in a way that limits transfer, for example by linking the comments to programme-level (rather than just module-level) learning outcomes, and illustrating how they might apply to other modules. Students themselves might recognize that improvement is not always instantaneous, and that they need to put in the “hard graft” to transfer feedback from one context to another (Carless, 2015). This might involve, for example, synthesizing feedback to draw out common themes across assignments. Finally, in terms of volition, educators' task is to employ sustainable feedback practices and ensure ample opportunities for dialogue, whilst also framing feedback in a motivating way such that improvement feels achievable for students. Students must in turn be willing to engage with the emotions that arise from receiving feedback, and adopt a positive, constructive “commitment to change” (Bing-You et al., 1997) in response to advice.

On a general level, a variety of interventions might help students to engage better with feedback, for example the use of self- and peer-assessment exercises, providing feedback literacy workshops, or withholding students' grades until after they have responded to the advice (Winstone et al., 2017). The evidence supporting the effectiveness of these kinds of interventions varies substantially, and all have strengths and limitations. But when choosing and designing any such intervention, the key focus should be not solely what the intervention should be, but rather, what skills it should ideally hone among students: fostering self-appraisal, assessment literacy, goal-setting and self-regulation, and/or engagement and motivation (Winstone et al., 2017).

Moreover, we propose that an equally important ingredient is a broader type of dialogue concerning the process and psychological experience of receiving feedback in general. It is apparent that most students in higher education have received little or no prior guidance on how to use feedback effectively (Weaver, 2006; Burke, 2009), and for this reason we must initiate and develop conversations with students about why engaging with feedback is important, what the barriers are, and the kinds of emotional responses we naturally have when faced with actual, implied, or anticipated criticism. These conversations should equip students to better anticipate and resist their own defensive reactions to feedback. With an increasingly diverse student body, these conversations might ideally also acknowledge that students' demographic and cultural backgrounds can shape their experiences of receiving feedback. One study, for example, qualitatively analyzed the personal reflections of Chinese postgraduates who were studying in the UK (Tian and Lowe, 2013). The data suggested that differences in academic cultures between China and the UK can create dissonance for students when receiving feedback. Many of the students reported feeling heartbroken and discouraged after receiving formative feedback, for instance, principally due to the sheer number of comments given. As they were not accustomed to receiving formative feedback, they interpreted their educators' extensive comments as a sign they were failing, rather than as a means of supporting their future improvement. These emotional reactions in turn limited the students' engagement with the feedback. This example clearly illustrates the importance of developing conversations around the experience of receiving feedback that are sensitive to cultural variations in students' expectations of education.

## Obstacles to responsibility-sharing

It would be naïve to imagine that making a case for responsibility-sharing, and setting out simple, descriptive assumptions of what it might involve, would be sufficient to actually deliver such a culture. Indeed, regardless of how we might undertake to foster responsibility-sharing between educators and students, several individual, institutional, and wider cultural obstacles might stand in the way. These obstacles need to be taken into consideration just as do the barriers to proactive recipience that we have already discussed.

One potential obstacle to responsibility-sharing is winning students' “buy-in” to and co-operation with this approach. This will undoubtedly be challenging within the apparently thriving consumer culture in higher education. We would therefore need to work hard to convince students of the rationale for demanding their proactive partnership in the feedback process. Given that the extent to which students' perception of the “value for money” of their course has declined in recent years (Neves and Hillman, 2017), it is important to convince students that playing such a proactive role is the *only* way they can ever get value for money in the domain of feedback. To this end, it will be crucial to frame proactive recipience as more than a purely academic skill, which helps students to understand why they earned the grades they earned. Rather, we must foster students' understanding that proactive recipience is a transferrable, sustainable, and lifelong skill that should support their employability and capacity to advance in their post-university careers. Some students, of course, will inevitably be more difficult than others to convince of the distal benefits of accepting their responsibilities in the present. But research tells us that students who naturally think about the future more than the past tend to be more engaged in and motivated by their academic achievements, and furthermore tend to perform more strongly (Husman and Lens, 1999; Horstmanshof and Zimitat, 2007). It is therefore important that we find ways to “sell” the long-term relevance of becoming effective consumers of feedback, not just the short-term benefits.

Students are not the only ones who might resist shifting toward a culture of responsibility-sharing—many educators will share students' skepticism. Indeed, educators' workloads are a key determinant of feedback practice (e.g., Hounsell, 2007), and the arguments we have made above imply that creating a culture of responsibility-sharing will involve even further investment from educators. As we have already noted, educators already view assessment and feedback as time-intensive, demanding activities, and so taking on new initiatives may seem implausible (Nicol, 2010). Why would an educator be willing to invest even more time and resources in undertaking activities to overcome the barriers we have discussed? We suggest that the investment of time in these activities in the short-term has the potential to secure the sustainability of feedback-related workload in the longer-term. If we can break down barriers that, in turn, equip and enthuse students to be proactive in seeking, creating, understanding and using feedback, then we as educators will no longer have to shoulder the overwhelming burden of responsibility by delivering more and more feedback with questionable effects.

Educators who accept the importance of responsibility-sharing may nevertheless feel that they are swimming against the tide, and fear that any efforts to shift toward such a culture will be seen as counter to achieving the high levels of student satisfaction against which teaching quality is increasingly assessed. Requiring students to play a role, even if we accept that this role is essential, can feel risky in this present context. But increasingly we do see more distal goals—beyond immediate student satisfaction—featuring in teaching quality metrics, such as measures of graduate employment and so-called “learning gain” (e.g., McGrath et al., 2015). With these more distally focused metrics in mind, it seems reasonable to conceive that a shift toward responsibility-sharing could still be quite consistent with institutional goals. That said, institutions themselves have roles to play in fostering supportive climates, wherein these important dialogues with students around responsibility-sharing can be initiated and developed. Likewise, the wider bodies responsible for quality assurance, including students' unions and policy makers, must recognize their own responsibilities for engendering cultures that promote and reward proactive recipience: cultures in which educators do not find it risky to expect students to share the responsibility of making feedback effective. Dialogue with these policy makers is needed.

Finally, we propose that individual educators' attempts to promote proactive recipience and responsibility-sharing are unlikely to have substantial effects unless students also receive congruent messages from the different educators with whom they have contact, and indeed from their institutions themselves. In short, achieving these goals will very much require a cultural shift as we have described it, rather than being fully achievable by dedicated individuals in isolation.

## Conclusion

Educators in higher education are reporting spiraling workloads as they attempt to offer students effective feedback with which they are satisfied. Yet it is increasingly apparent not only that this approach is unsustainable for educators, but that it is highly unlikely to ever be effective for students either. No matter how quick, how detailed, or how high-quality the feedback our students receive, feedback can never be effective unless they use it, and therefore educators alone do not have the power to ensure that feedback is impactful. Sharing responsibilities in the specific domain of feedback is therefore essential.

We have argued that numerous barriers can stand in the way of students engaging proactively with the feedback they receive, and the approach to responsibility-sharing set out in this paper assumes that both students and educators have equal but partly distinct roles in tackling these barriers. This approach further assumes an inherent degree of interdependence: neither students nor educators can necessarily fulfill all their roles without the other party doing the same. Developing a culture of this kind is, we believe, a sustainable way of shifting the burden of responsibility, rather than only shifting the blame.

## Author contributions

RN and NW both made substantial contributions to the preparation and writing of this paper.

### Conflict of interest statement

The authors declare that the research was conducted in the absence of any commercial or financial relationships that could be construed as a potential conflict of interest.
